# Supplementary material for: Dynamic interaction of BRCA2 with telomeric G-quadruplexes underlies telomere replication homeostasis
Source: Nat Commun. 2022 Jun 13;13:3396. doi: 10.1038/s41467-022-31156-z (PMC9192595; doi:10.1038/s41467-022-31156-z)
Supplement: Supplementary file 1 — Supplementary Information [file 41467_2022_31156_MOESM1_ESM.pdf]

Supplementary Information for:

Dynamic interaction of BRCA2 with telomeric G-quadruplexes underlies telomere replication homeostasis

Junyeop Lee[1§], Keewon Sung[2§], So Young Joo[1], Jun-Hyeon Jeong[1], Seong Keun Kim[2], and Hyunsook Lee[1*]*

[1]*Department of Biological Sciences & IMBG, Seoul National University, Seoul 08826, South Korea*

[2]*Department of Chemistry, Seoul National University, Seoul 08826, South Korea*

[§]These authors contributed equally.

*Co-correspondence: H.L. (HL212@snu.ac.kr) & S.K.K. (seongkim@snu.ac.kr)

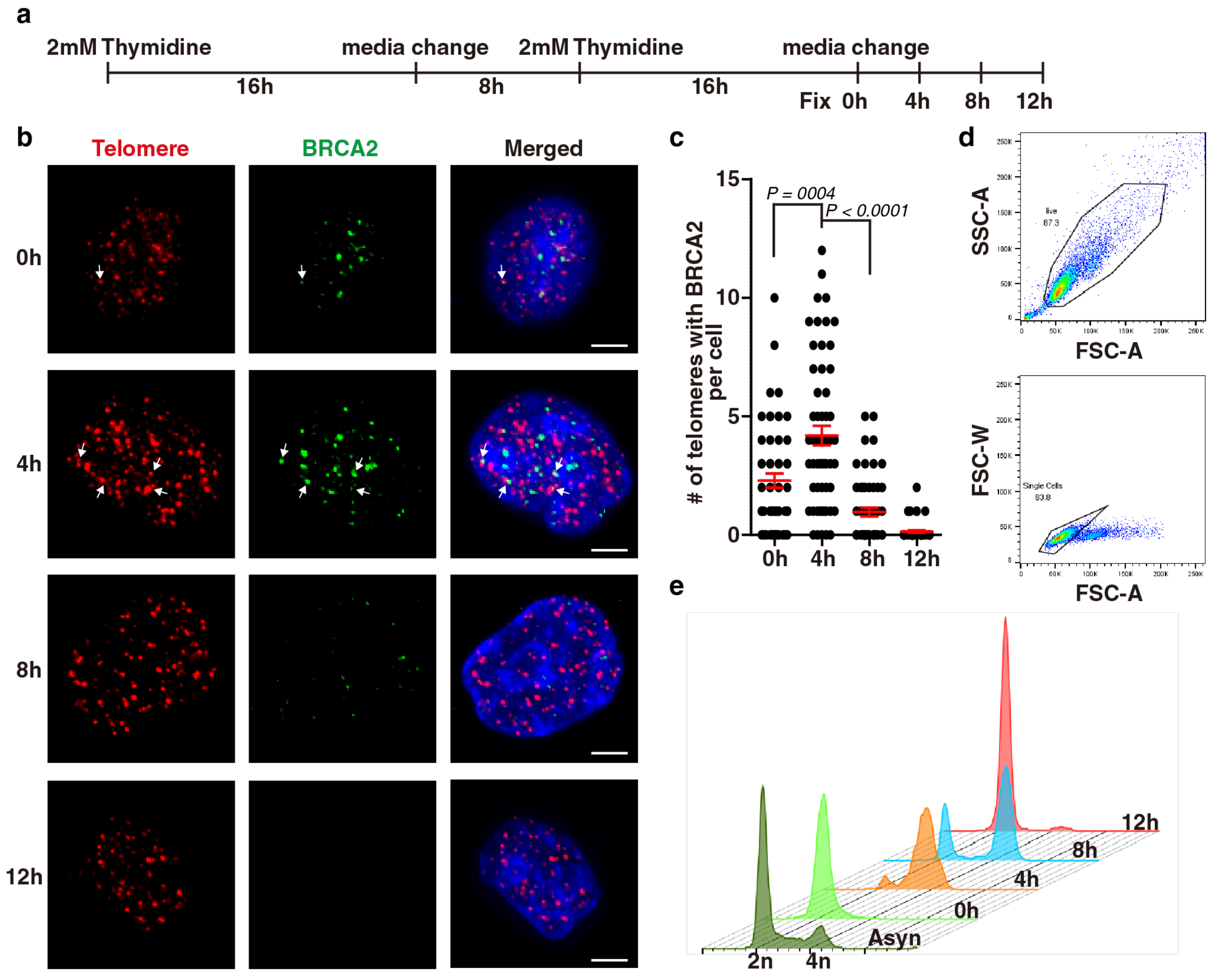

Supplementary Figure 1. BRCA2 localizes to telomeres in S-phase. **(a)** Schematic workflow of the experiment. NFLAP-BRCA2 HeLa cells[1] were synchronized in G1/S using a thymidine double block and then washed to release into the cell cycle. Cells were then fixed at indicated time points for immunostaining or for flow cytometry. **(b)** Immunostaining coupled with FISH analysis. Immunostaining with anti-GFP antibody was performed to detect BRCA2 (NFLAP-BRCA2), followed by denaturation and FISH analysis with a PNA-labeled telomere probe. Green, BRCA2; Red, telomere. White scale bar, 5 µm. **(c)** Scoring of telomeres positive of BRCA2 after immunoFISH. Number of cells analyzed: 0 h, n = 55; 4 h, n = 58; 8h, n = 56; 12 h, n = 62. All *P* values were obtained with the two-sided Student's *t*-test (mean ± s.e.m.). **(d)** Gating of live cells for flow cytometry analysis for cell cycle. Forward scatter area (FSC-A)/side scatter area (SSC-A) and forward scatter area (FSC-A)/ forward scatter width (FSC-W) are shown. **(e)** Flow cytometry analysis of propidium iodide staining.

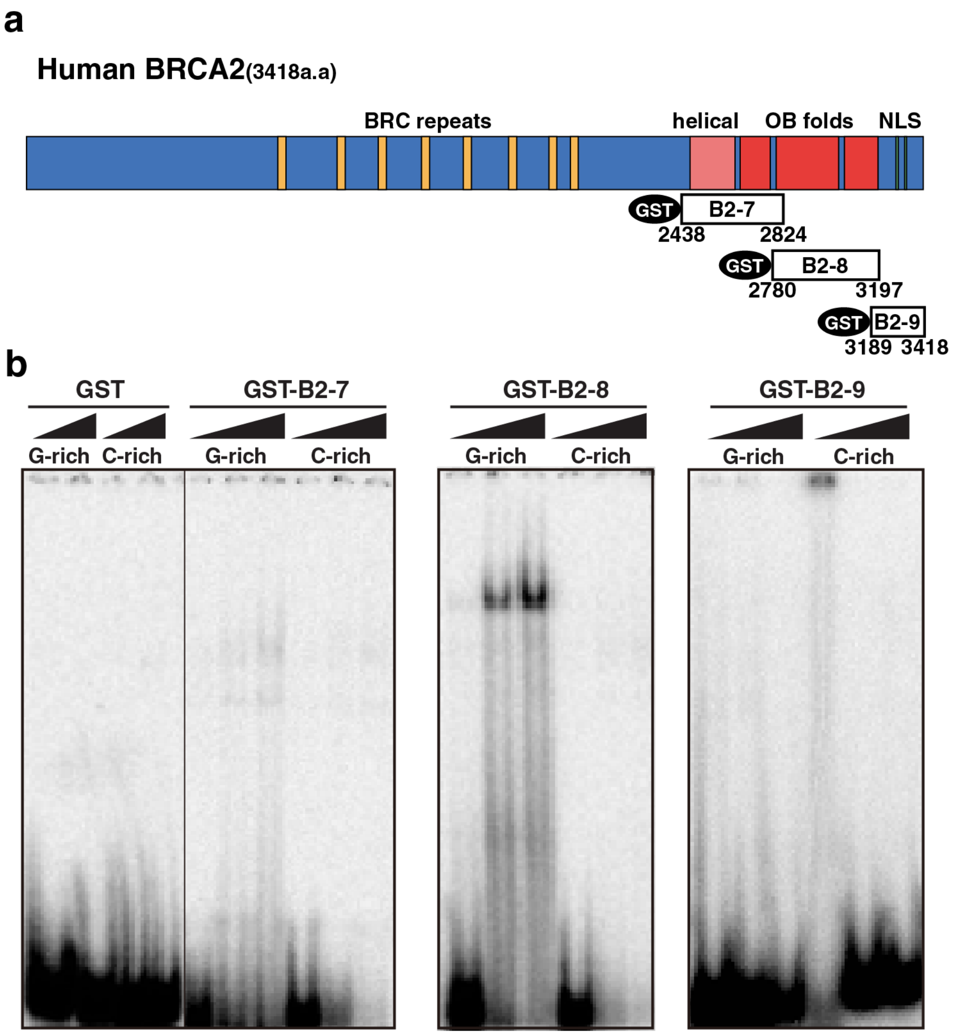

Supplementary Figure 2. The OB-folds-containing fragment of BRCA2 binds to the telomere G-rich strand. **(a)** Schematic illustration of hBRCA2. GST-tagged B2-7 (hBRCA2 2438-2824), B2-8 (hBRCA2 2780-3197), and B2-9 (hBRCA2 3189-3418),–which include part of the BRCA2 ssDNA binding domain (DBD), were purified from *E. coli*. **(b)** Only the GST-B2-8, containing the OB-folds, bound to the telomeric G-rich strand (5'-GCC CGC GGT TAG GGT TAG GGT TAG GGT TAG GGG TGA ATT C-3') in a concentration-dependent manner. GST-B2-8 does not bind to the C-rich complementary strand (5'-GAA TTC ACC CCT AAC CCT AAC CCT AAC CCT AAC CGC GGG C-3'). The reaction buffer contained 150 mM KCl. The experiment was repeated two times independently..

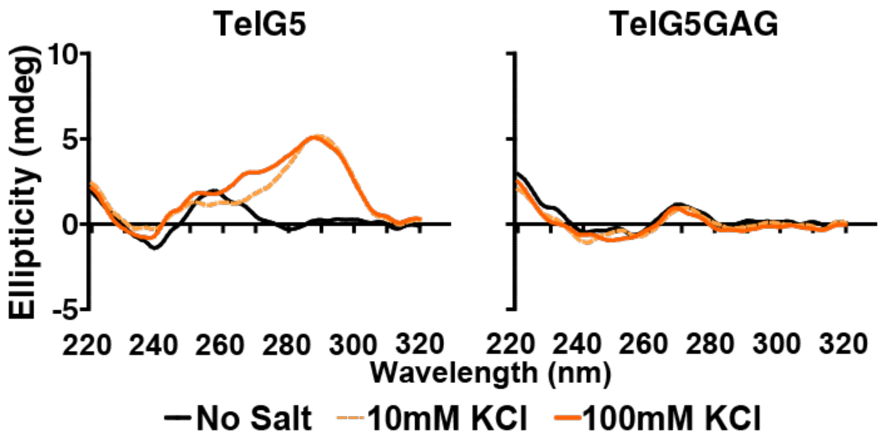

Supplementary Figure 3. Conformations of TelG5 and TelG5GAG revealed by CD spectra. CD (Circular Dichroism) spectra of TelG5 and TelG5GAG were analyzed under three conditions; no salt (black), 10 mM KCl (yellow, dashed), and 100 mM KCl (orange). In contrast to TelG5, in which the ellipticity increases substantially at higher concentration of KCl, no significant change was observed for TelG5GAG, indicative of the unfolded structure.

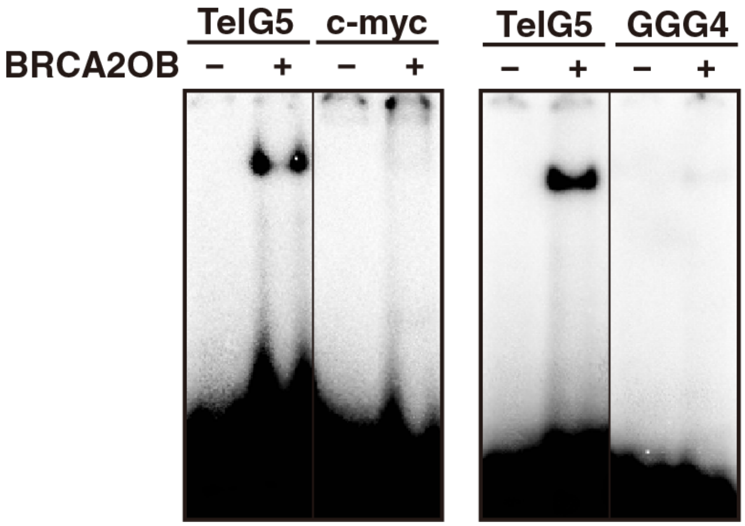

Supplementary Figure 4. BRCA2OB specifically interacts with telomeric G4. Recombinant BRCA2OB was incubated with radiolabeled TelG5, G4-forming *c-MYC* promoter (c-myc), or an artificial sequence designed to fold into G4, and subjected to EMSA. BRCA2OB specifically binds to TelG5. The reaction buffer contained 10 mM Na^+ but no K^+ ions. The experiments were repeated three times independently.

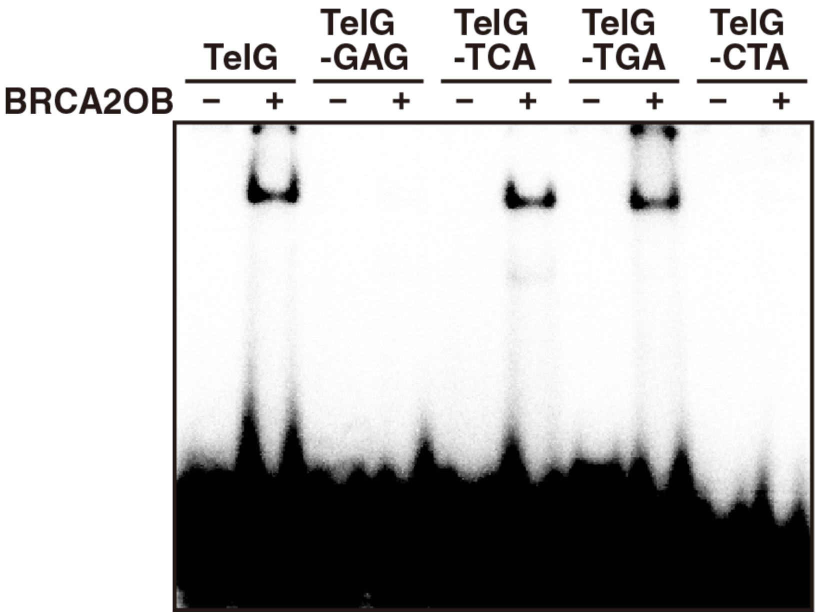

Supplementary Figure 5. Differential binding profile of BRCA2OB to telomere variants. EMSA was performed with several telomere variant repeats. TelG-TCA, -TGA, and -CTA represent telomere variants of wild-type TelG, where TTA is substituted to TCA, TGA, and CTA, respectively (Supplementary Table 1). TelG-GAG is the unfolded mutant control, as the GGG repeat is substituted with GAG. The reaction buffer contained 10 mM Na^{+}. The experiment was repeated three times independently.

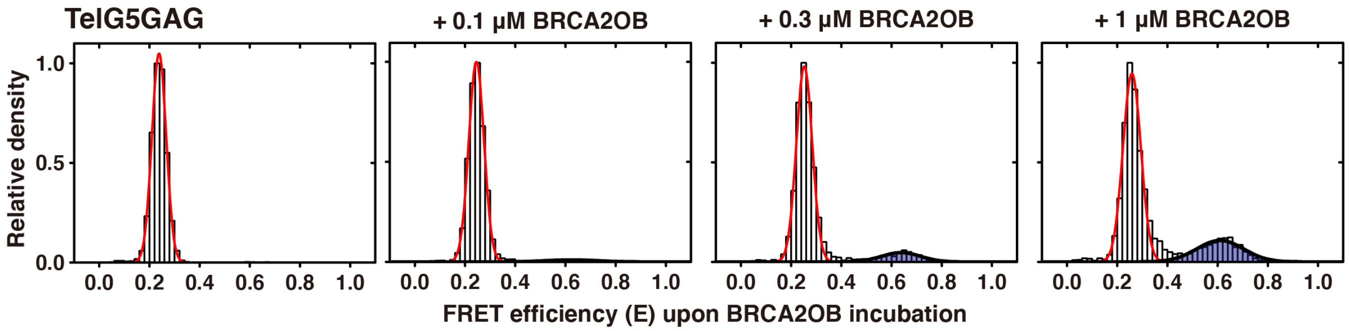

Supplementary Figure 6. Interaction of BRCA2OB with TelG5GAG. Gaussian fitted smFRET histograms of TelG5GAG with increasing concentrations of BRCA2OB (color-coded as in **Fig. 2g**). Negligible binding (< 3%) was observed in 0.1 μM BRCA2OB, and the bound fraction remained marginal (~20%) even after increasing BRCA2OB concentration to 1 μM.

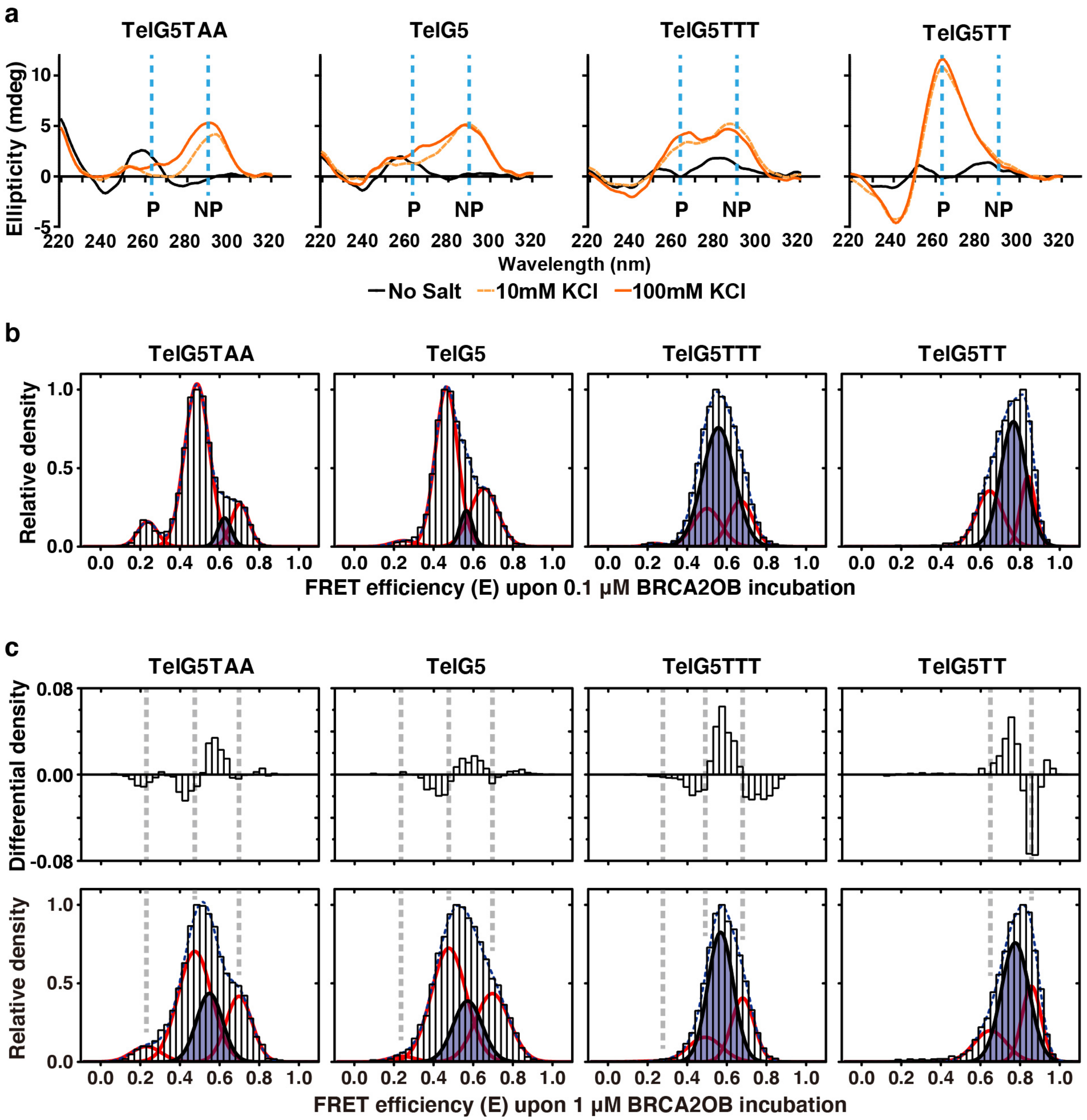

Supplementary Figure 7. Mode of BRCA2OB-binding to telomeric G4 variants. **(a)** CD spectra of various G4 constructs. CD spectra of four different G4-forming oligonucleotides (TelG5TAA, TelG5, TelG5TTT, and TelG5TT) in no salt (black), 10 mM KCl (yellow, dashed), and 100 mM KCl (orange). **(b)** smFRET histograms of the four G4 constructs upon incubation with 0.1 μM BRCA2OB with Gaussian fits colored as in Fig. 2g. **(c)** Differential density histograms (upper) and smFRET histograms upon incubation with 1 μM BRCA2OB (lower) of the four G4 constructs. Note the emergence of a new peak between the NP and P conformational ones after BRCA2OB addition.

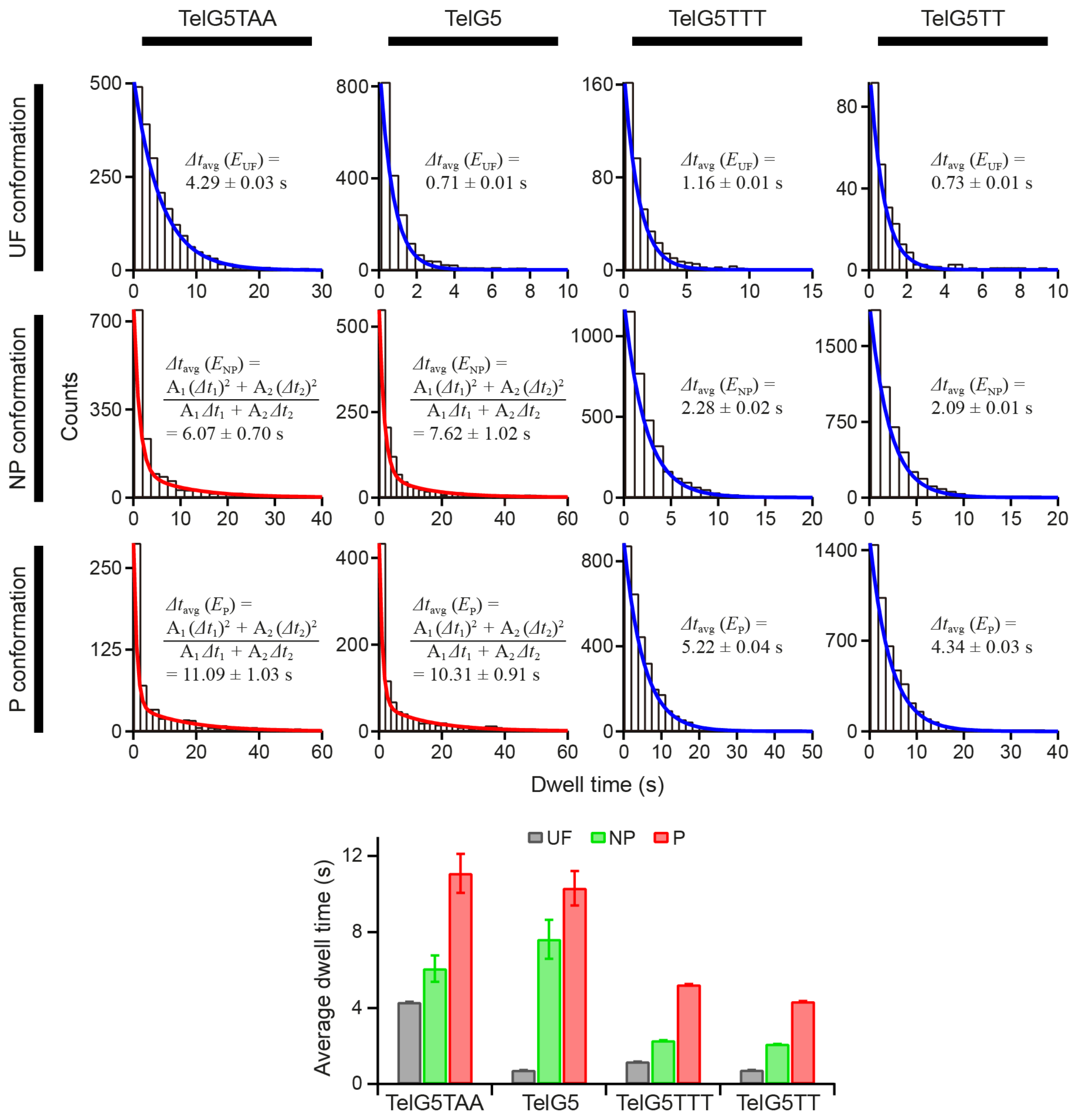

Supplementary Figure 8. Dwell time analysis of the four G4 constructs. Dwell time distributions of the four G4 constructs (TelG5TAA, TelG5, TelG5TTT, and TelG5TT) in UF (top), NP (middle), and P (bottom) conformations (without BRCA2OB). The average dwell time (Δt_{avg}) was extracted from the single-exponential fit (blue) of the UF conformation for all constructs, and NP and P conformations for TelG5TTT and TelG5TT. The dwell time distributions of NP and P conformations for TelG5TAA and TelG5 are better described using a bi-exponential fit (red), consistent with a previous report[2]; the average dwell time corresponds to the amplitude-weighted average value calculated by the equation at each plot. (Δt_{avg} ± s.d. from each fit shown). Dwell-time data were collected from >300 molecules from three independent experiments. The bar graph at the bottom summarizes the average dwell time of each conformation (Δt_{avg} ± s.d. from the fit).

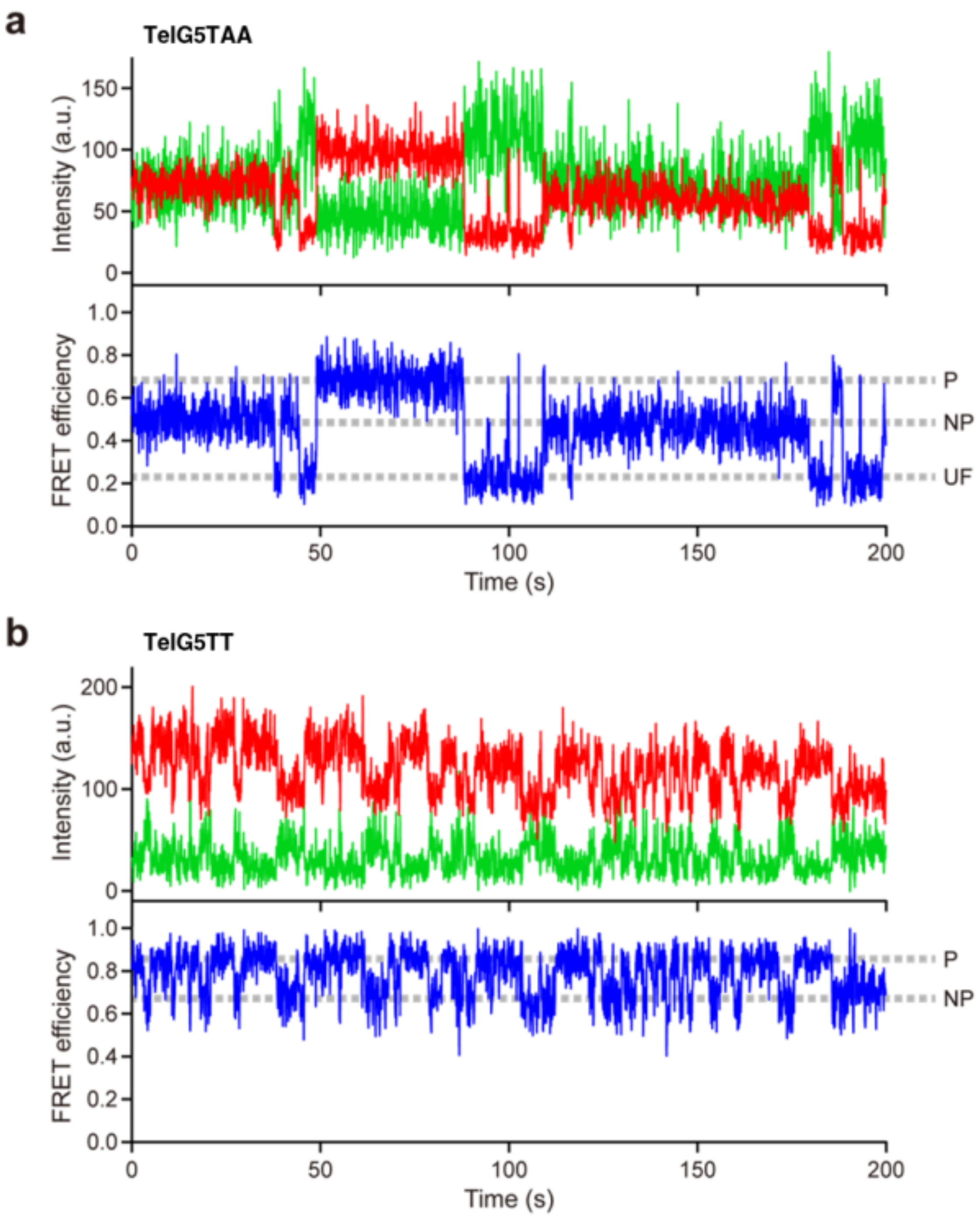

Supplementary Figure 9. Representative time trajectories of TelG5TAA and TelG5TT. (a) A representative time trajectory of TelG5TAA dynamics showing structural rearrangements that largely transits through UF conformation. **(b)** A representative time trajectory of the TelG5TT molecule which transitions directly between NP and P conformations without passing through the UF conformation. Both trajectories were obtained in the absence of BRCA2OB.

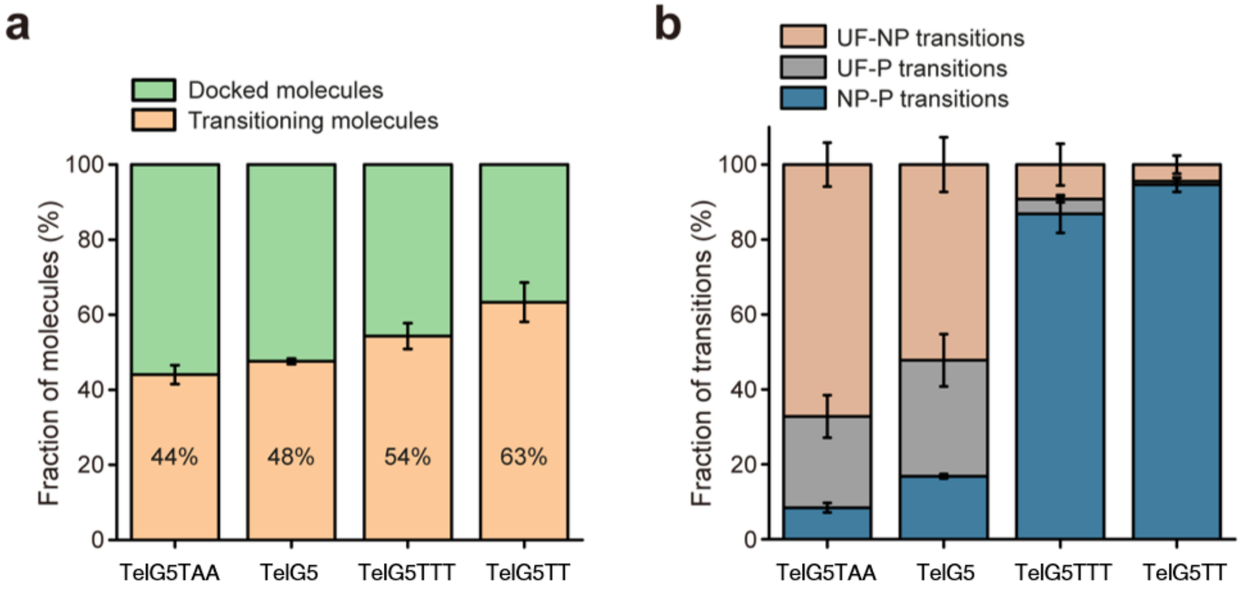

Supplementary Figure 10. Quantification of molecular transition density. **(a)** Fraction of G4 molecules showing time trajectories which are stably docked in a single FRET state (green) or transition between multiple FRET states (orange). **(b)** Relative abundance of the transitions between UF and NP conformations (UF → NP and NP → UF; beige), UF and P conformations (UF → P and P → UF; gray), and NP and P conformations (NP → P and P → NP; blue) quantified from the transition density plot in **Fig. 3d**. The density of the NP-P direct transition in **Fig. 3f** was calculated by multiplying the fraction of transitioning molecules in (a) by the fraction of NP-P transitions in (b). Mean ± s.d. from three independent experiments are shown.

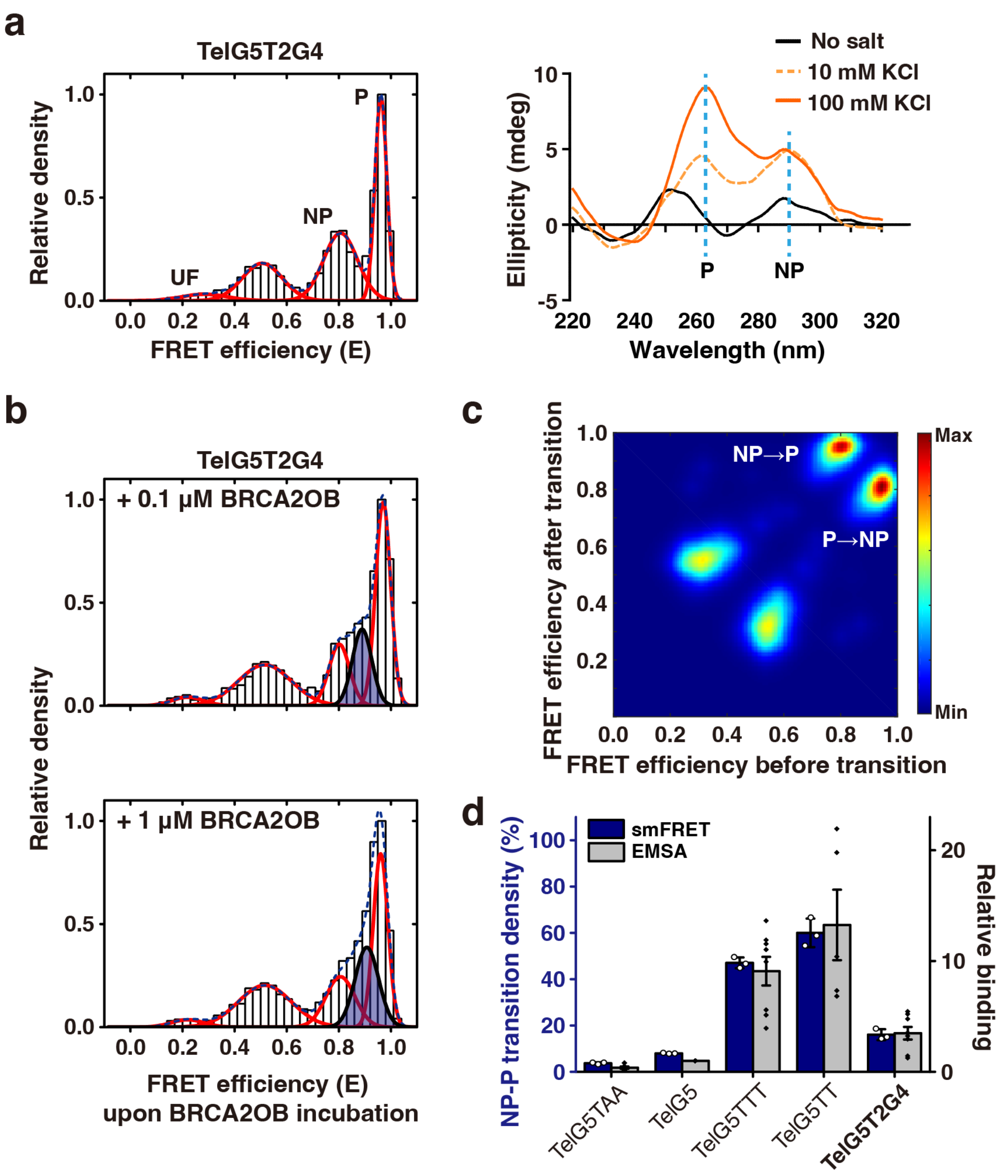

Supplementary Figure 11. Conformational dynamics and BRCA2OB-binding characteristics of TelG5T2G4. **(a)** A Gaussian fitted smFRET histogram of TelG5T2G4 alone (left) and its CD spectra under three conditions (right); no salt (black), 10 mM KCl (yellow, dashed), and 100 mM KCl (orange). The FRET states are heterogeneous including an unidentified state (*E*~0.5), possibly due to the formation of four stacks of G-tetrads (i.e., the TTGGGG repeat in TelG5T2G4). **(b)** Gaussian fitted smFRET histograms of TelG5T2G4 upon incubation with 0.1 μM (top) and 1 μM (bottom) of BRCA2OB (color-coded as in **Fig. 2g**). **(c)** A transition density plot representing relative abundance of each transition among the four FRET states. **(d)** A plot comparing the density of direct NP-P transitions (mean ± s.d., n = 3) calculated from the smFRET results (blue bars, left axis) and relative binding of BRCA2OB (mean ± s.e.m., n ≥ 5) determined from the EMSA data (gray bars, right axis). The plot is identical to **Fig. 3f**, but the values of the TelG5T2G4 construct are added. All experiments were repeated independently.

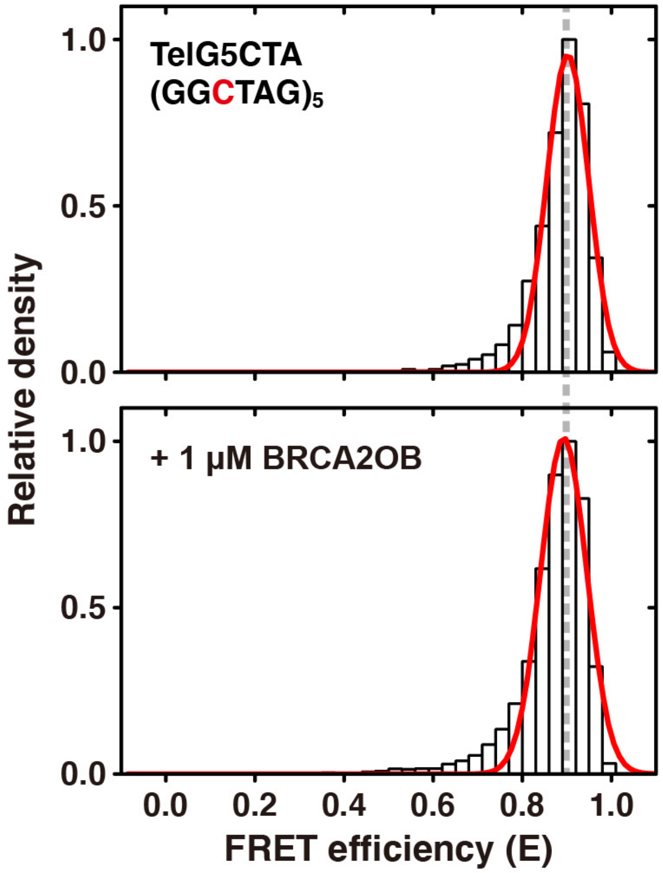

Supplementary Figure 12. Conformation and BRCA2OB-binding characteristics of TelG5CTA. smFRET histograms of TelG5CTA (TTA loop mutated to CTA) alone (upper) and after incubation with 1 μM of BRCA2OB (lower) are shown. Unlike other G4 substrates (i.e., TelG5, TelG5TAA, TelG5TTT, TelG5TT, and TelG5T2G4), TelG5CTA molecules exhibited a single FRET peak (E ~ 0.9). Upon BRCA2OB incubation, the FRET histogram barely changed, consistent with the above EMSA data (Supplementary Fig. S5). These results support that BRCA2OB does not bind to stably folded G4 structures.

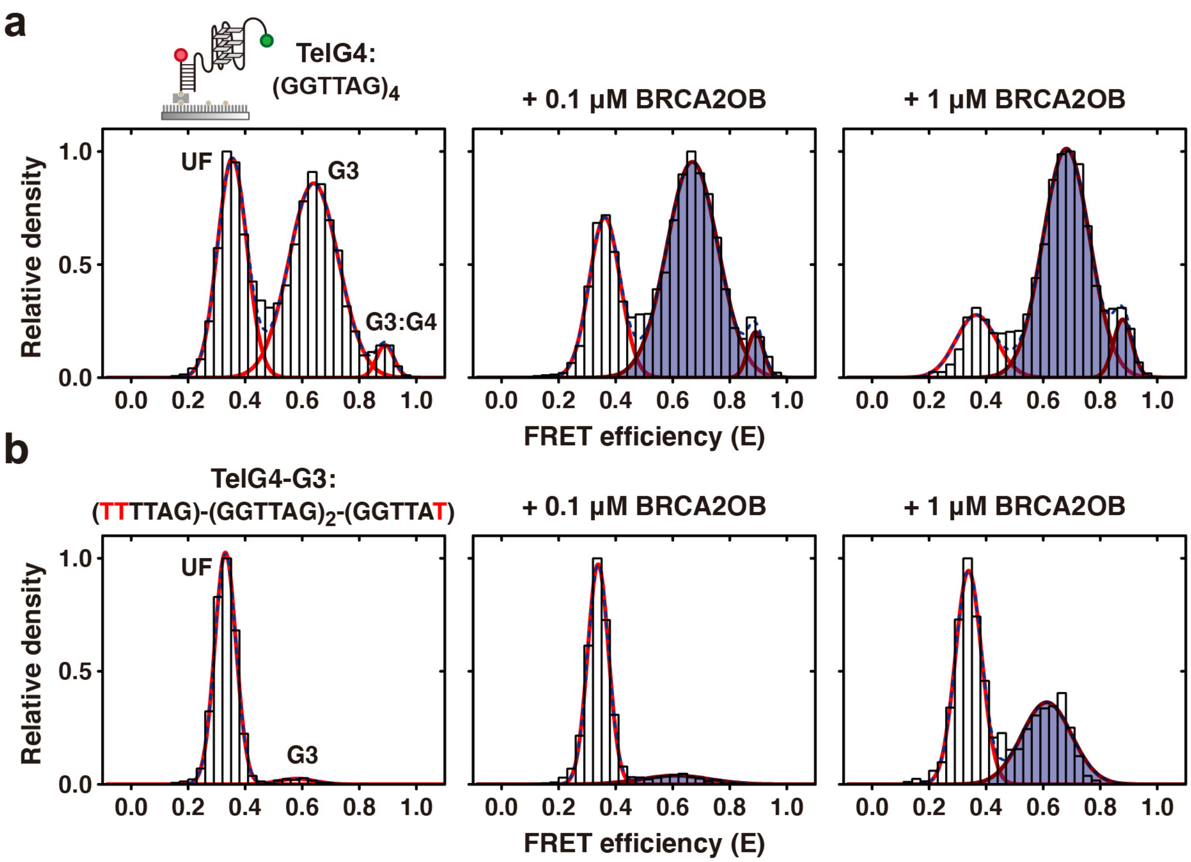

Supplementary Figure 13. Characterization of G3-derived structures and their binding towards BRCA2OB. **(a)** smFRET histograms of TelG4 (left) which contains three GGG triplets (cartoon at the top) similar to TelG5-G3 (Fig. 4), alone (left) and in the presence of 0.1 μM (middle) and 1 μM (right) of BRCA2OB. Three FRET states were observed, as seen for TelG5-G3. FRET values were slightly changed compared to TelG5-G3, due to differences in construct length. Upon addition of BRCA2OB, the middle-FRET state becomes stronger. **(b)** smFRET histograms of TelG4-G3, where G bases in the tail sequence other than the three GGG triplets are replaced with T, alone (left) or upon incubation with BRCA2OB at 0.1 μM (middle) and 1 μM (right). For TelG4-G3, a single folded state ($E \sim 0.6$) was identified with low population (~4%), indicating that the middle-FRET state corresponds to the G3 structure. BRCA2OB effectively captured the minor population of G3, resulting in a bound fraction of ~40% with 1 μM BRCA2OB. Given that the only difference between TelG4 and TelG4-G3 is the presence of G bases in the tail sequence, the high-FRET state of TelG4 was assigned as a G3:G4 structure involving the tail that forms through interactions of G3 with extra G bases in the tails. On the basis of structural homology between TelG4 and TelG5-G3, both of which comprise three GGG triplets and the same tail sequences (i.e., GGTTA and TTAG at the 5' and 3' end, respectively), the high-FRET state of TelG5-G3 also represents the tail-associated G3:G4 conformation, while the middle-FRET state for TelG5-G3 likely consists of not only G3 but a "loop-associated" G3:G4 structure that may be assembled by the interaction between the G3 and remaining G bases in the long loop sequence (Fig. 4).

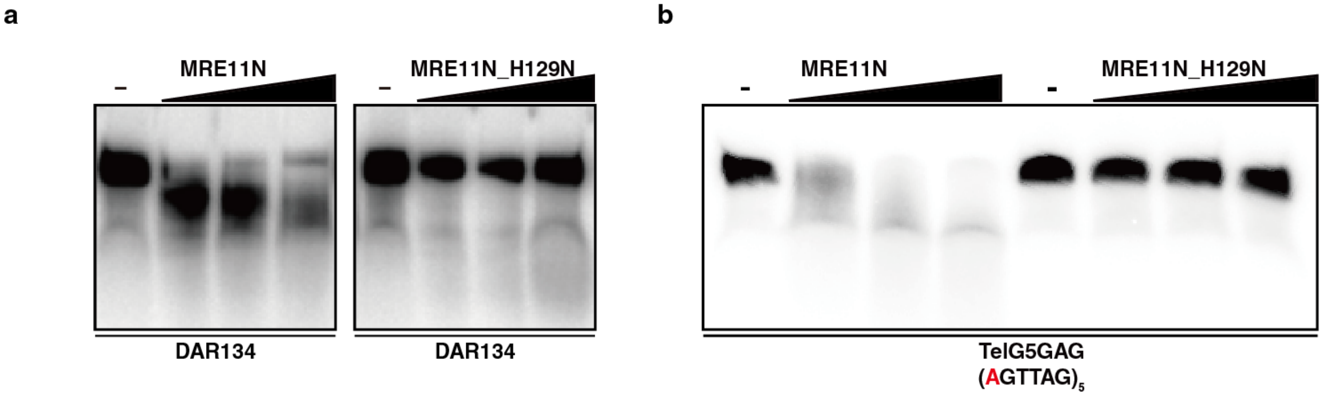

Supplementary Figure 14. Nuclease activity of the purified MRE11N. **(a)** Denaturing PAGE (polyacrylamide gel electrophoresis) of a hairpin-like secondary structured DNA (DAR134)[3], before or after incubation with MRE11N (left) or the nuclease-dead form Mre11N_H129N (right). Recombinant MRE11N cleaved DAR134 in a concentration-dependent manner, whereas MRE11N_H129N did not. **(b)** Denaturing PAGE of the effect of MRE11N on unstructured single-strand DNA (TelG5GAG). MRE11N degraded the substrate completely through its 5’ to 3’ exo-nuclease activity, while MRE11N_H129N did not. The result is the representative of three independent experiments.

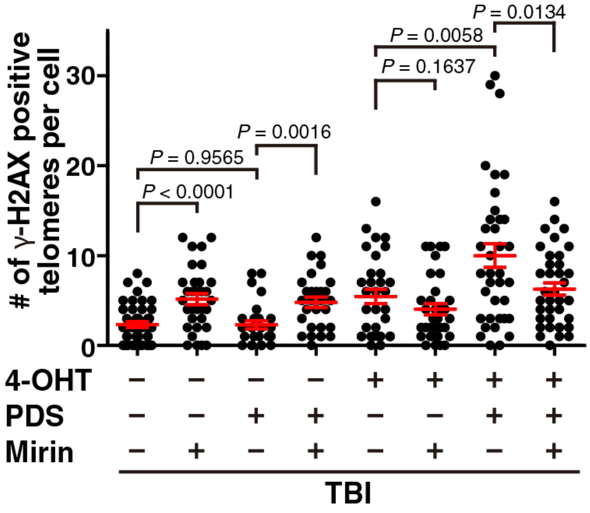

Supplementary Figure 15. Frequency of telomere damage with or without 4-OHT, PDS, or Mirin. Mirin, the Mre11 inhibitor, showed consistent results with Mre11 depletion by lenti-shMre11 treatment (Fig. 7b). The number of cells analysed: NT (no treatment), n = 46; + Mirin, n = 32; + PDS, n = 25; + Mirin + PDS, n = 27; + 4-OHT, n = 32; + Mirin + 4-OHT, n = 33; + PDS + 4-OHT, n = 37; + Mirin + PDS + 4-OHT, n = 38. All *P* values were obtained with the two-sided Student's t-test (mean ± s.e.m.).

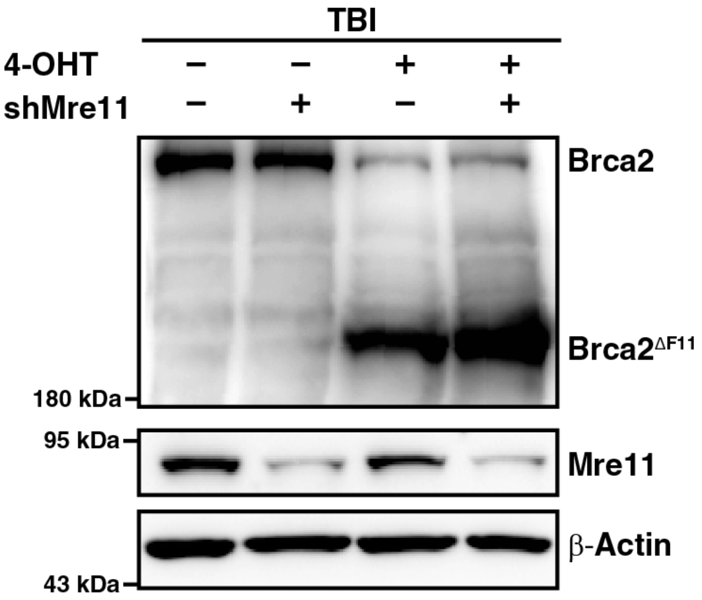

Supplementary Figure 16. Western blot analysis of decreased Brca2 and Mre11. Western blot analysis to assess the efficiency of Brca2 depletion and Mre11 after 4-OHT treatment or lenti-*shMre11* transduction in **Fig.7 a-b**. Beta-actin was used as a loading control. The result is the representative of two independent experiments.

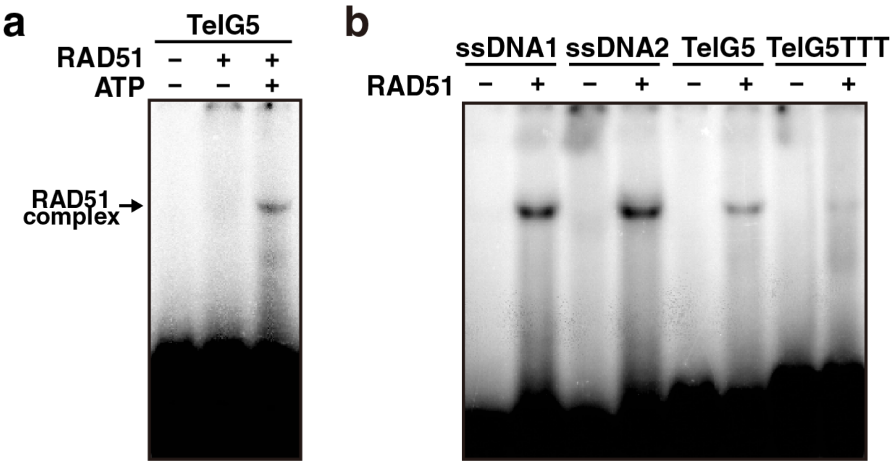

Supplementary Figure 17. Characteristics of RAD51 binding to DNA. EMSA was performed with recombinant RAD51. **(a)** Binding of RAD51 (250 nM) to TelG5 was observed in the presence of 2 mM ATP. **(b)** Comparison of RAD51 binding to ssDNA1 (CGGACTCAGATCTCG-AGCTCGCATGCCTAT), ssDNA2 (AGCTCGCATGCCTATTGGATCCAAAGAGAG), TelG5, and TelGTTT. The result is the representative of three independent experiments.

Supplementary Table 1. Sequences of oligonucleotides used in EMSA and nuclease assay.

Name	Sequence
TelG3	5’ – GGT TAG GGT TAG GGT TAG – 3’
TelG4	5’ – GGT TAG GGT TAG GGT TAG GGT TAG – 3’
TelG5	5’ – GGT TAG GGT TAG GGT TAG GGT TAG GGT TAG – 3’
TelG6	5’ – GGT TAG GGT TAG GGT TAG GGT TAG GGT TAG GGT TAG – 3’
TelC5	5’ – CTA ACC CTA ACC CTA ACC CTA ACC CTA ACC – 3’
TelG5GAG	5’ – AGT TAG AGT TAG AGT TAG AGT TAG AGT TAG – 3’
TelG5TTT	5’ – GGT TTG GGT TTG GGT TTG GGT TTG GGT TTG – 3’
TelG5T2G4	5’ – GGT TGG GGT TGG GGT TGG GGT TGG GGT TGG – 3’
TelG5TAA	5’ – GGT AAG GGT AAG GGT AAG GGT AAG GGT AAG – 3’
TelG5TT	5’ – GGT TTG GGT TGG GTT GGG TTG GGT TTG – 3’
TelG5-G3	5’ – GGT TAG GGT TAG GGT TAG TGT TAG GGT TAG – 3’
TelG3GGG	5’ – GGG TTA GGG TTA GGG TTA GGG – 3’
c-myc	5’ – TTG AGG GTG GGG AGG GTG GGG AAT T– 3’
GGG4	5’ – GAA CTG GGA CTG GGA TAG GGC TAG GGA AGC– 3’
TelG	5’ – GTT AGG GTT AGG GTT AGG GTT AGG GTT AG – 3’
TelG-TCA	5’ – GTC AGG GTC AGG GTC AGG GTC AGG GTC AG – 3’
TelG-TGA	5’ – GTG AGG GTG AGG GTG AGG GTG AGG GTG AG – 3’
TelG-CTA	5’ – GCT AGG GCT AGG GCT AGG GCT AGG GCT AG – 3’
TelG-GAG	5’ – GTT AGA GTT AGA GTT AGA GTT AGA GTT AG – 3’
ssDNA1	5’ – CGG ACT CAG ATC TCG AGC TCG CAT GCC TAT – 3’
ssDNA2	5’ – AGC TCG CAT GCC TAT TGG ATC CAA AGA GAG – 3’

Supplementary Table 2. Sequences of oligonucleotides used in single-molecule FRET assays.

Name	Sequences
TelG5	5' – GCG TGG CAC CGG TAA TAG GAG ATA GGA GAG GTT AGG GTT AGG GTT AGG GTT AGG GTT AGG GTT AG /**3AmMO**/ – 3'
TelG5TAA	5' – GCG TGG CAC CGG TAA TAG GAG ATA GGA GAG GTA AGG GTA AGG GTA AGG GTA AGG GTA AG **/3AmMO/** – 3'
TelG5TTT	5' – GCG TGG CAC CGG TAA TAG GAG ATA GGA GAG GTT TGG GTT TGG GTT TGG GTT TGG GTT TG **/3AmMO/** – 3'
TelG5TT	5' – GCG TGG CAC CGG TAA TAG GAG ATA GGA GAG GTT TGG GTT GGG TTG GGT TGG GTT TG /**3AmMO**/ – 3'
TelG5GAG	5' – GCG TGG CAC CGG TAA TAG GAG ATA GGA GAA GTT AGA GTT AGA GTT AGA GTT AGA GTT AG /**3AmMO**/ – 3'
TelG5T2G4	5' – GCG TGG CAC CGG TAA TAG GAG ATA GGA GAG GTT GGG GTT GGG GTT GGG GTT GGG GTT GG /**3AmMO**/ – 3'
TelG5CTA	5' – GCG TGG CAC CGG TAA TAG GAG ATA GGA GAG GCT AGG GCT AGG GCT AGG GCT AGG GCT AG /**3AmMO**/ – 3'
TelG5-G3	5' – GCG TGG CAC CGG TAA TAG GAG ATA GGA GAG GTT AGG GTT AGG GTT AGT GTT AGG GTT AG /**3AmMO**/ – 3'
TelG5TTT-G3	5' – GCG TGG CAC CGG TAA TAG GAG ATA GGA GAG GTT TGG GTT TGG GTT TGT GTT TGG GTT TG /**3AmMO**/ – 3'
TelG4	5' – GCG TGG CAC CGG TAA TAG GAG ATA GGA GAG GTT AGG GTT AGG GTT AGG GTT AG /**3AmMO**/ – 3'
TelG4-G3	5' – GCG TGG CAC CGG TAA TAG GAG ATA GGA GAT TTT AGG GTT AGG GTT AGG GTT AT /**3AmMO**/ – 3'
stem	5' – /**5AmMC6**/ TCT CCT ATC TCC TAT TAC CGG TGC CAC GC /**Biotin**/ – 3'

"3AmMO" and "5AmMC6" refer to amino-modification at the 3' and 5' end, respectively, for dye labeling.

References

1. Choi, E. et al. BRCA2 Fine-Tunes the Spindle Assembly Checkpoint through Reinforcement of BubR1 Acetylation. *Dev Cell* **22**, 295-308 (2012).
2. Lee, J.Y., Okumus, B., Kim, D.S. & Ha, T. Extreme conformational diversity in human telomeric DNA. *Proc Natl Acad Sci U S A* **102**, 18938-43 (2005).
3. Park, Y.B., Chae, J., Kim, Y.C. & Cho, Y. Crystal structure of human Mre11: understanding tumorigenic mutations. *Structure* **19**, 1591-602 (2011).
