# Supplementary material for: Dynamic interaction of BRCA2 with telomeric G-quadruplexes underlies telomere replication homeostasis
Source: Nat Commun. 2022 Jun 13;13:3396. doi: 10.1038/s41467-022-31156-z (PMC9192595; doi:10.1038/s41467-022-31156-z)
Supplement: Supplementary file 3 — Reporting Summary [file 41467_2022_31156_MOESM3_ESM.pdf]

nature portfolio

Corresponding author(s): Seong Keun Kim and Hyunsook Lee

Last updated by author(s): May 12, 2022

Reporting Summary

Nature Portfolio wishes to improve the reproducibility of the work that we publish. This form provides structure for consistency and transparency in reporting. For further information on Nature Portfolio policies, see our Editorial Policies and the Editorial Policy Checklist.

Statistics

For all statistical analyses, confirm that the following items are present in the figure legend, table legend, main text, or Methods section.

n/a	Confirmed	
☐	☒	The exact sample size (*n*) for each experimental group/condition, given as a discrete number and unit of measurement
☐	☒	A statement on whether measurements were taken from distinct samples or whether the same sample was measured repeatedly
☐	☒	The statistical test(s) used AND whether they are one- or two-sided<br>*Only common tests should be described solely by name; describe more complex techniques in the Methods section.*
☒	☐	A description of all covariates tested
☒	☐	A description of any assumptions or corrections, such as tests of normality and adjustment for multiple comparisons
☐	☒	A full description of the statistical parameters including central tendency (e.g. means) or other basic estimates (e.g. regression coefficient) AND variation (e.g. standard deviation) or associated estimates of uncertainty (e.g. confidence intervals)
☐	☒	For null hypothesis testing, the test statistic (e.g. *F*, *t*, *r*) with confidence intervals, effect sizes, degrees of freedom and *P* value noted<br>*Give P values as exact values whenever suitable.*
☒	☐	For Bayesian analysis, information on the choice of priors and Markov chain Monte Carlo settings
☒	☐	For hierarchical and complex designs, identification of the appropriate level for tests and full reporting of outcomes
☒	☐	Estimates of effect sizes (e.g. Cohen's *d*, Pearson's *r*), indicating how they were calculated

Our web collection on statistics for biologists contains articles on many of the points above.

Software and code

Policy information about availability of computer code

Data collection	Multi Gauge v3.0 software was used to measure labeled DNA intensities.<br>An open-source program (Single v1.0.4) was used to collect the single-molecule fluorescence signals, which can be downloaded from https://github.com/pjb7687/single/
Data analysis	GraphPad Prism v5, FlowJo v10.7.1, IDL (version 7.0.0), MATLAB R2016b, Igor Pro 6.3, and open-source codes (Raw data analysis version 1.0.0) from https://github.com/Ha-SingleMoleculeLab/Raw-Data-Analysis/, and open-source software (vbFRET_nov12) from http://vbfret.sourceforge.net/ were used. All the other auxiliary codes used for smFRET data analysis are available upon request to the corresponding authors.

For manuscripts utilizing custom algorithms or software that are central to the research but not yet described in published literature, software must be made available to editors and reviewers. We strongly encourage code deposition in a community repository (e.g. GitHub). See the Nature Portfolio guidelines for submitting code & software for further information.

Data

Policy information about availability of data

All manuscripts must include a data availability statement. This statement should provide the following information, where applicable:

- Accession codes, unique identifiers, or web links for publicly available datasets
- A description of any restrictions on data availability
- For clinical datasets or third party data, please ensure that the statement adheres to our policy

Uncropped scans of all Western blots, gels, and raw data for graphs are provided in the Source Data file. The authors declare that all data supporting the finding of this study is available within this article, supplementary information or from the corresponding author upon reasonable request.

Field-specific reporting

Please select the one below that is the best fit for your research. If you are not sure, read the appropriate sections before making your selection.

☒ Life sciences ☐ Behavioural & social sciences ☐ Ecological, evolutionary & environmental sciences

For a reference copy of the document with all sections, see nature.com/documents/nr-reporting-summary-flat.pdf

Life sciences study design

All studies must disclose on these points even when the disclosure is negative.

Sample size	For electrophoretic mobility shift assay, nuclease assay, and dot blotting, more than three independent experiments were performed. The presented data are the representative of multiple experiments.<br>For single-molecule experiments, we used data from >2000 molecules to build each FRET histogram, and >200 single-molecule FRET time trajectories were used for the dwell-time and transition-density analyses. The sample size was determined by single-molecule density in the field of view, which is limited by the diffraction limit and microscopy setup. Each experiment was repeated for 2–5 times to ensure reproducibility.<br>For immunofluorescence assay, three independent experiments were performed. More than 30 cells were analyzed so that the sample size meets the requirement of the central limit theorem.
Data exclusions	For the smFRET experiments, these data were excluded: 1) if the Cy5 signal was lacked (i.e., FRET efficiency = 0) which corresponds to a signal from the Cy3-only-labeled-molecule; 2) if two or more Cy3 (or Cy5) dye molecules were observed, which are identified by multi-step photobleaching or abnormally high level of total intensity (Cy3 + Cy5) after gamma correction that deviates from a single-molecule intensity distribution.
Replication	All data were confirmed with more than two independent experiments. The results were robustly reproduced.
Randomization	For immunofluorescence assay, cell images were randomly scored to avoid bias.
Blinding	In immunofluorescence, the samples were blindly scored to avoid bias. All experiments were performed following the same protocol and the data analyzed with identical criteria.

Reporting for specific materials, systems and methods

We require information from authors about some types of materials, experimental systems and methods used in many studies. Here, indicate whether each material, system or method listed is relevant to your study. If you are not sure if a list item applies to your research, read the appropriate section before selecting a response.

Materials & experimental systems

n/a	Involved in the study	
☐	☒	Antibodies
☐	☒	Eukaryotic cell lines
☒	☐	Palaeontology and archaeology
☒	☐	Animals and other organisms
☒	☐	Human research participants
☒	☐	Clinical data
☐	☒	Dual use research of concern

Methods

n/a	Involved in the study	
☒	☐	ChIP-seq
☐	☒	Flow cytometry
☒	☐	MRI-based neuroimaging

Antibodies

Antibodies used	Sheep polyclonal anti-mouse Brca2 antibodies were home made: generated by injection of recombinant mouse Brca2 protein (3,107–3,303 amino acids) into sheep and purified as referenced in the manuscript. The following antibodies were purchased and used in the indicated dilution ratio: anti-MBP mAb (E8032, New England BioLabs, 1:10000); anti-β-actin rabbit mAb (A700-057, Bethyl Laboratories, 1:1000); anti-Mre11 (4895S, Cell signaling Technology, 1:1000); anti-gamma-H2AX (ser139) rabbit mAb (9718S, Cell Signaling Technology, 1:200); anti-TRF1 mouse mAb (TRF-78) (ab10579. Abcam, 1:200); and anti-Rad51 rabbit pAb (Ab-1) (PC130, Calbiochem, 1:500).
Validation	All experiments were optimized and confirmed suitable with minimum backgrounds prior to actual experiments: Anti-Mre11-, anti-Anti-MBP- and anti-beta-actin antibodies were found suitable for western blotting in mouse cells. Anti-gamma-H2A.X-, anti-TRF1-, and anti-Rad51 antibodies were confirmed suitable for immunofluorescence in mouse cells. Results were confirmed by adequate controls and compared with the data from the manufacturer's website and also with the data provided from the previous manuscripts.<br>Home-made anti-sheep polyclonal mouse Brca2 antibodies were confirmed by comparing with the previous experiments (Choi et al;. Dev. Cell, 2012; Min et al. J Biol Chem. 2012 & Kwon et al. FEBS J. 2019). These manuscripts are referenced in the manuscript.

Eukaryotic cell lines

Policy information about cell lines

Cell line source(s)	Telomerase-negative and Brca2-depletion-inducible (TBI) mouse fibroblast : MEFs from Brca2F11/F11; mTR-/-; Cre-ERTM mouse were immotalized with introduction of SV40-LT (Kwon et al. FEBS J. 2019). This manuscript was referenced. NFLAP-BRCA2 HeLa cell line was provided from Mark Petronczki (Lekomtsev et al., J. Cell. Sci. 2010), which we successfully used in previous reports (Choi et al., Dev. Cell. 2012). 293T cells (CRL-3216) and HeLa cells (CRM-CCL-2) were purchased originally from ATCC and maintained in the lab.
Authentication	Brca2 depletion from the Telomerase-negative and Brca2-depletion-inducible mouse fibroblast (TBI) was confirmed by genomic PCR as described previously (Kwon et al., 2019). Expression of BRCA2 in NFLAP-BRCA2 HeLa was confirmed by Western blot with anti-BRCA2 antibodies and anti-GFP antibodies. Cells were maintained with media containing G418, as previously described by Petronczki (Lecomtsev et al., 2012). This manuscript is cited.
Mycoplasma contamination	All cells used were regularly tested for mycoplasma contamination. When cells were fixed and stained with DAPI, we confirmed that there were no visual sign of mycoplasma contamination.
Commonly misidentified lines (See ICLAC register)	There are no commonly misidentified cell lines in the study. All cell lines used in the study are identified and appropriately cited.

Dual use research of concern

Policy information about dual use research of concern

Hazards

Could the accidental, deliberate or reckless misuse of agents or technologies generated in the work, or the application of information presented in the manuscript, pose a threat to:

No	Yes	
☒	☐	Public health
☒	☐	National security
☒	☐	Crops and/or livestock
☒	☐	Ecosystems
☒	☐	Any other significant area

Experiments of concern

Does the work involve any of these experiments of concern:

No	Yes	
☒	☐	Demonstrate how to render a vaccine ineffective
☒	☐	Confer resistance to therapeutically useful antibiotics or antiviral agents
☒	☐	Enhance the virulence of a pathogen or render a nonpathogen virulent
☒	☐	Increase transmissibility of a pathogen
☒	☐	Alter the host range of a pathogen
☒	☐	Enable evasion of diagnostic/detection modalities
☒	☐	Enable the weaponization of a biological agent or toxin
☒	☐	Any other potentially harmful combination of experiments and agents

Flow Cytometry

Plots

Confirm that:

☒ The axis labels state the marker and fluorochrome used (e.g. CD4-FITC).

☒ The axis scales are clearly visible. Include numbers along axes only for bottom left plot of group (a 'group' is an analysis of identical markers).

☒ All plots are contour plots with outliers or pseudocolor plots.

☒ A numerical value for number of cells or percentage (with statistics) is provided.

Methodology

Sample preparation	For analyzing cell cycle profiles of each time point, cells were seeded and arrested in G1/S phase by double-thymidin block. Then, the cells were released to S phase and fixed by 70% ethanol at each time point. The fixed cells were treated with RNase A and DNA stained with propidium iodide (PI).
Instrument	BD FACSCanto II
Software	FlowJo v10.7.1
Cell population abundance	We counted 10,000 PI stained cells for each experiment.
Gating strategy	We analyzed cells with FSC-A/SSC-A and FSC-A/FSC-W to gate live single cells then measured the DNA content.

☒ Tick this box to confirm that a figure exemplifying the gating strategy is provided in the Supplementary Information.
